# Bezoars: Culprits of gastrointestinal obstruction that may lead to surgical intervention and potentially surgical complications

**DOI:** 10.1002/ccr3.8126

**Published:** 2023-12-05

**Authors:** Shannon D. Powell, Nicholas Pereira

**Affiliations:** ^1^ Department of Pediatrics Saint James School of Medicine Arnos Vale Saint Vincent and the Grenadines; ^2^ Department of Pediatrics South Texas Health System Children's Hospital Edinburg Texas USA

**Keywords:** abdominal pain, bezoars, gastrointestinal tract, intestinal obstruction

## Abstract

**Key Clinical Message:**

Bezoars can cause gastrointestinal obstruction and may require surgery. Surgery carries the risk of complications. Medical professionals should perform detailed history in diet and behaviors for patients presenting with abdominal pain to identify risk factors for bezoars, then educate these patients and their families about risks and complications of bezoars.

**Abstract:**

Bezoars are solid masses of undigested material that can cause obstruction of the gastrointestinal tract. There are different types of bezoars; phytobezoar, trichobezoar, pharmacobezoar, lactobezoar, and bezoars containing tissue paper or polystyrene foam. This case report is of a 13‐year‐old Hispanic male who suffered a postsurgical complication after removal of bezoar. He had a past surgical history of appendectomy and presented to the hospital with a 1‐day history of right lower quadrant abdominal pain associated with fever and diarrhea. X‐radiation images and computed tomography scans aided in the diagnosis of pelvic abscess as a complication of postsurgical enterotomy and closure of the enterotomy to remove bezoar from the small bowel. The initial bezoar removal and the postsurgical complication of pelvic abscess resulted in the patient staying for 19 days in the hospital. At discharge, the patient and his guardian were advised to follow up with the patient's primary care physician and surgical team. The patient made an uneventful recovery. He did not experience any long‐term complications and fully recovered. This report demonstrates that although bezoars are rare, they can cause significant obstruction of the gastrointestinal tract leading to the need for management, such as surgery, which carries its own risks. It is important to note that the postsurgical complication of pelvic abscess can occur due to surgery itself and not because of bezoar specifically. Abdominal surgery in general poses the risk of pelvic abscess. The consideration is to expectantly decrease the occurrence of bezoars so that consequently there will not be a need for surgery in removal of bezoars due to obstruction. The effects of bezoars can be prevented through educating the community and addressing underlying psychiatric disorders.

## Graphical abstract

CT scan of the abdomen and pelvis. Arrow points to intestinal bezoar.

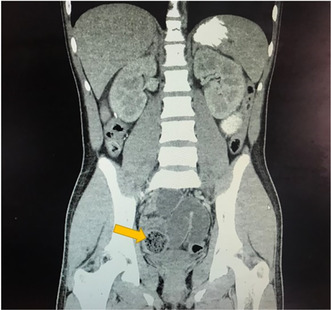

## 1 INTRODUCTION

Bezoars are solid collections of tightly packed material that are composed of undigested and sometimes, inedible material, that becomes fixed or stuck in the gastrointestinal tract. The term “bezoar” comes from the word “badzehr,” which is Arabic for “antidote”.^1^ Historically, animal bezoars were thought to have medicinal properties to cure many human diseases and reverse poisons. The types or kinds of diseases that bezoars were used to treat were just about anything as the bezoars were also thought to have magical properties going back over 2000 years ago.^2^

The first bezoar was found in the stomach of a goat and had a hard, green concrete texture.^2^ Some bezoars were decorated with gold and jewels to be carried as charms with the purpose of keeping any illness, venom, and poison away.^2^ This was until the theory was tested by surgeon Ambroise Paré in the 1500s, who debunked the idea that animal bezoars were antidotes or had healing properties.^3^ He did so by having a thief who was sentenced to death, receive a poison and then a bezoar, with the condition that if he survives, he would be able to go free. The thief died within 7 h of receiving the poison, and Paré concluded that the theory was false.^3^ The term “bezoar” has been modernly adapted to refer to a medical condition.

The classification of bezoar is based on its contents. The most common type is phytobezoar, which makes up about 40% of reported bezoars.^3^ It is composed of indigestible fruit and vegetable particles, including peels and seeds. A subset of phytobezoar is diospyrobezoar, which is more specific to indigestible material from persimmons, particularly the skin tannin, which agglutinates in stomach acid and binds protein very strongly.^4^ Other types of bezoars include trichobezoars (contains hair), pharmacobezoars (contains hardened material of different kinds of drugs), and lactobezoars (contains milk protein). Bezoars can also be composed of materials, such as tissue paper and polystyrene foam products (ex: foam cups).^5^

Bezoars are not spoken about very often as they are relatively rare. Many times, they are found incidentally. However, the impact that they may have can be quite significant. The purpose of this article is to provide more awareness on bezoars and to shed much more light on why it is important to prevent them, with ways to do so. There are articles available that relate to the topic of bezoars, but very few speak about the need to enhance familiarity with the topic as it relates to improving patient education.

This case report describes a 13‐year‐old Hispanic male who presented with acute right lower quadrant abdominal pain and was diagnosed with pelvic abscess 3 weeks status post small bowel enterotomy for removal of bezoar.

## 2 CASE REPORT

**FIGURE 1:**
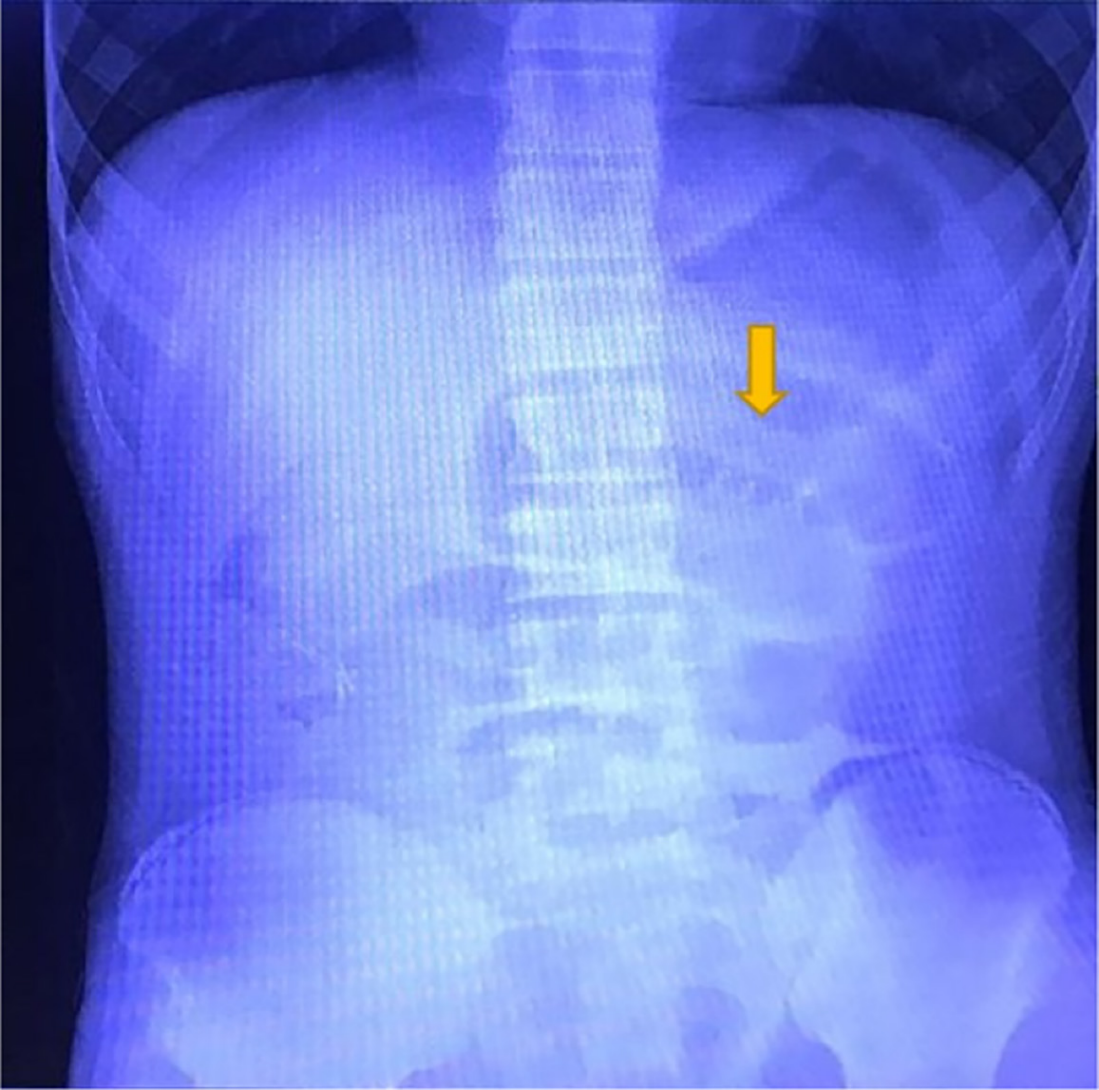
X‐ray of the abdomen (supine, anteroposterior view) revealing small bowel distention in the mid‐abdomen (arrow).

**FIGURE 2:**
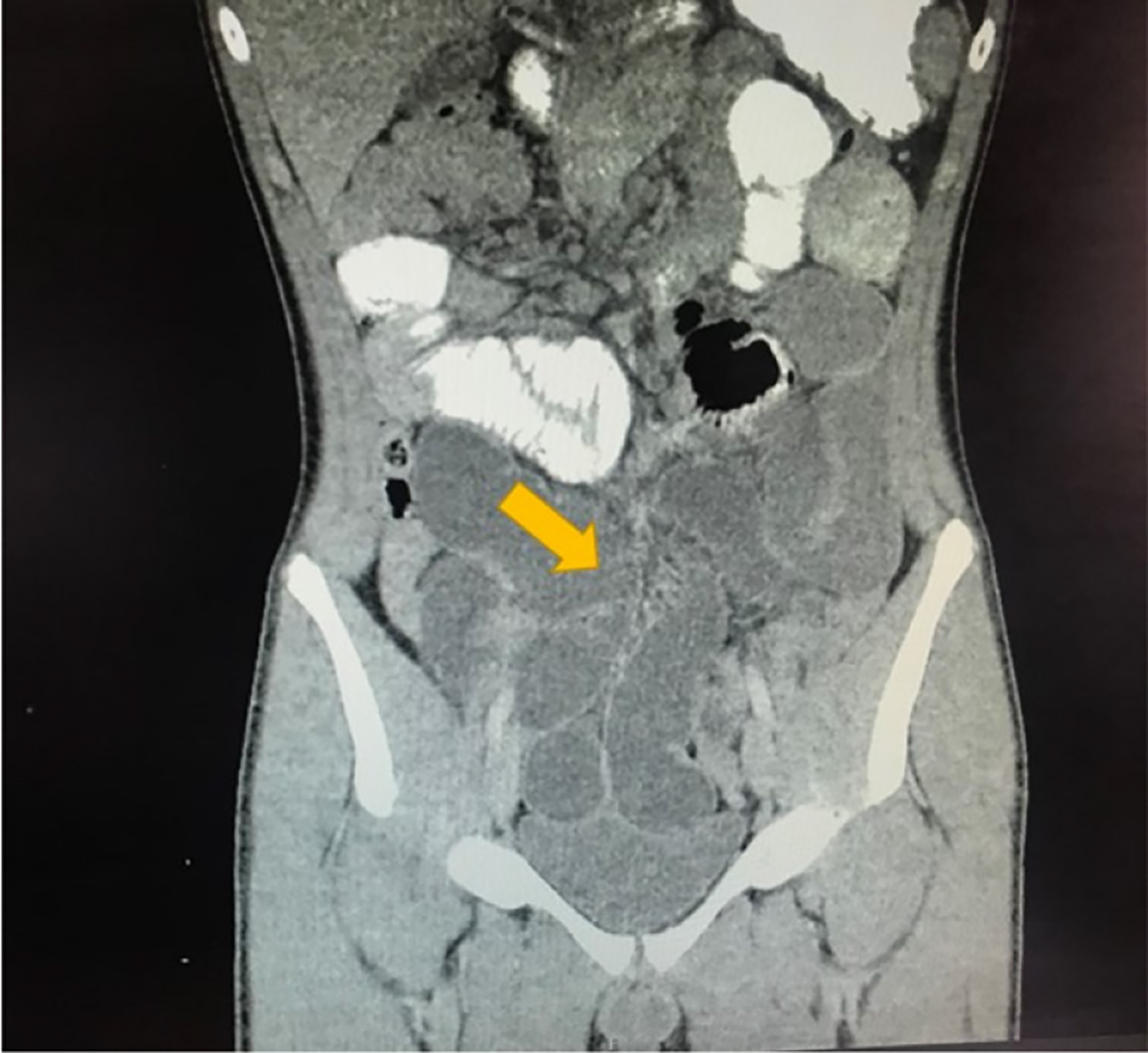
CT scan of the abdomen and pelvis with contrast showing multiple dilated loops of small bowel throughout the abdomen (arrow).

**FIGURE 3:**
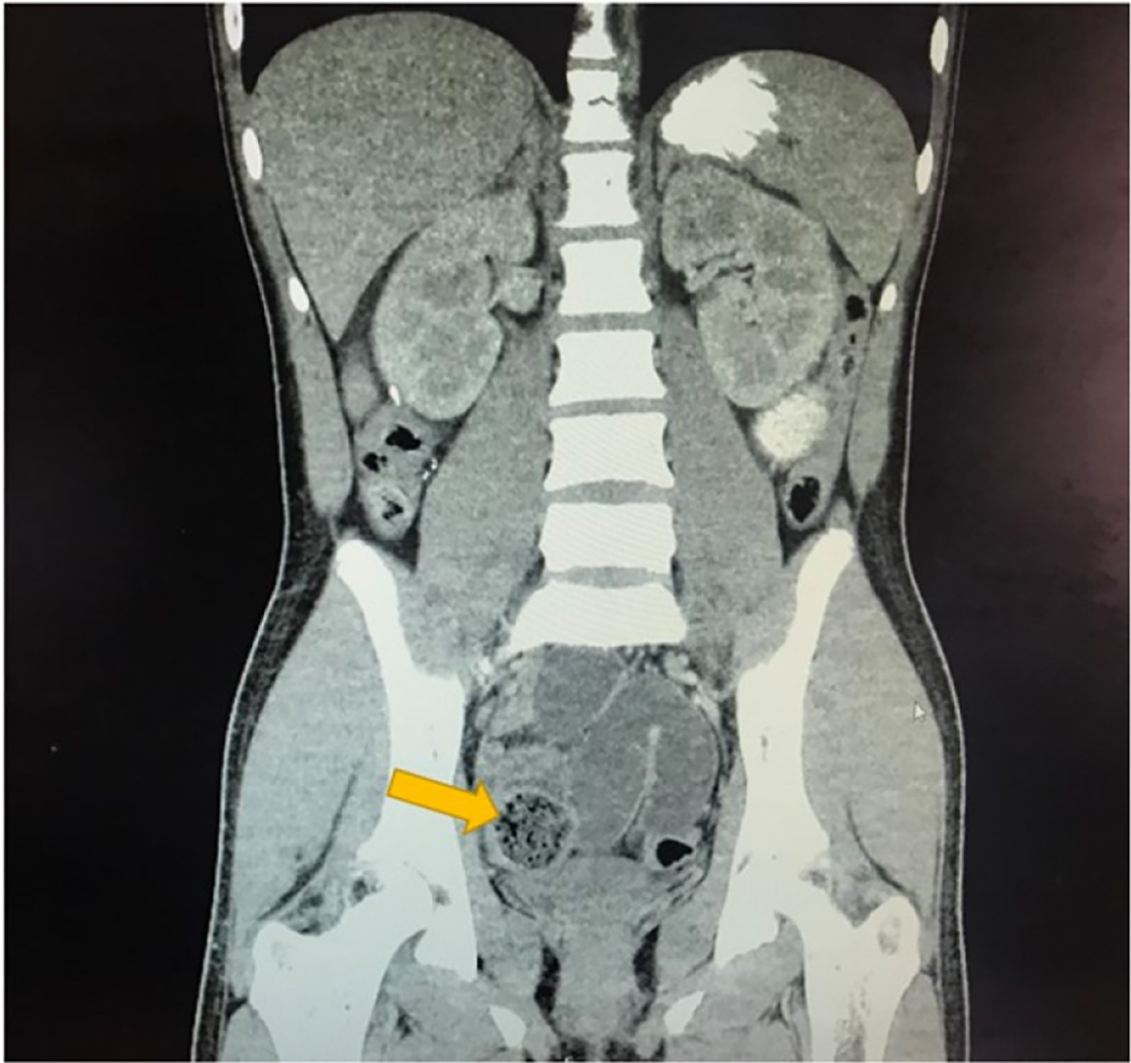
CT scan of the abdomen and pelvis with contrast showing a focus of fecalized small bowel contents (arrow).

A 13‐year‐old Hispanic male with a history of appendectomy over six years prior to this case visit was brought by his mother to the hospital due to generalized abdominal pain. The patient's prior appendectomy was due to appendicitis and no complications were stated. An X‐radiation (x‐ray) of the abdomen taken due to his generalized abdominal pain revealed small bowel distention in the mid‐abdomen (Figure 1). This was followed by a computed tomography (CT) scan of the abdomen and pelvis with contrast, which showed multiple dilated loops of small bowel throughout the abdomen suggestive of small bowel obstruction (Figure 2). A transition point was visualized in the right lower quadrant near the pelvis. At the transition point, there was a focus of fecalized small bowel contents (Figure 3). Additionally, it was noted that there were changes consistent with prior appendectomy.

The patient's previous appendectomy in the possibility of malrotation was discussed and the decision was made to schedule the patient for a minimally invasive attempt for surgery. The patient was taken for surgery and had a diagnostic laparoscopic enterotomy and closure that resulted in removal of bezoar from the ileum, which was causing intestinal obstruction. This ruled out the possible diagnosis of malrotation. The bezoar specimen was noted to be of a large amount of white fibrous material of unclear etiology that did not appear to be digestible. The patient was admitted for 10 days which included postoperative recovery and monitoring.

The patient was readmitted 3 weeks later due to presenting symptoms after the surgery; he was brought by his mother to the hospital due to a 1‐day history of fever, right lower quadrant abdominal pain, and diarrhea. The patient had three episodes of diarrhea the night before presenting to the hospital. While taking the history, the patient revealed that he had an ongoing habit of eating small amounts of toilet paper. His vitals were temperature of 39.5°C, blood pressure of 116/64 mmHg, heart rate of 102 beats per minute, respiratory rate of 20 breaths per minute, and oxygen saturation of 100% on nasal cannula at a rate of 3 liters per minute. Upon physical examination, the patient had tenderness to palpation of the abdomen, especially in the right lower quadrant. Laboratory findings included increased white blood cell count of 14.40 × 10^9^/L, decreased hemoglobin and hematocrit levels of 88 g/L and 0.277, respectively, and increased platelet count of 960 × 10^9^/L.

A repeated x‐ray of the abdomen was done that showed clips over the right mid/lower abdomen consistent with the enterotomy for the bezoar removal. This x‐ray did not demonstrate significant air‐fluid collection levels within the small or large bowel. The following repeat‐CT scan of the abdomen and pelvis with contrast, revealed a complex finding within the pelvis that demonstrated an air‐fluid level. This led to the diagnosis of pelvic abscess.

The patient was started on intravenous meropenem/normal saline, 1 g/50 mL every 8‐hour interval. A CT‐guided percutaneous drainage catheter placement of the pelvic abscess was performed the next day. Five days later a follow‐up CT scan of the abdomen and pelvis with contrast was done and showed that the previously noted abnormal air‐fluid collection in the pelvis had improved significantly and nearly resolved. The patient tolerated the medications well and improved with no rashes secondary to medications.

The patient was admitted for 9 days and was discharged with oral antibiotics, cefdinir 300 mg one capsule by mouth every 12 h for 7 days, and metronidazole 500 mg one tablet by mouth every 8 h for 7 days. On the day of discharge, the patient was stable and had made an uneventful recovery. His vitals had normalized with resolved fever, he had no bloody stools or edema, and he had good oral intake. Patient and guardian were advised to follow‐up with patient's primary care physician in 7 days in conjunction with follow‐up with surgical team. The patient did not experience any long‐term complications and fully recovered.

## 3 DISCUSSION

This case report demonstrates that bezoars can cause significant intestinal obstruction. It was the culprit that caused the patient's need for surgery. The occurrence of a bezoar in this patient is somewhat shocking, not just because he did not have considerable risk factors, but because bezoars are rare. This patient had no diagnosed behavioral disorder or developmental delay. However, he did have iron deficiency anemia, which can cause the habit of eating non‐nutritional, inedible material, such as soil, clay, ice, and even tissue paper, in a condition known as pica.

Bezoars can cause gastric or intestinal obstruction depending on the location of the bezoar. Bezoar incidence is variable among studies with one study reporting 0.43% over a 4‐year period, another reporting 0.43% over a 7‐year period, and yet another study reporting an incidence of 0.068% over a 20‐year period.^6^ Gastric bezoars have a reported frequency of less than 0.5%, while bezoars in the small intestine have a reported frequency of 0.4%–4.8%.^6^

Although there is no consensual pathogenesis of bezoars, contributing factors of gastric bezoars are low gastric acidity, impaired digestion, and loss of normal function of the pyloric sphincter.^3^ Delayed gastric emptying, such as in diabetes mellitus, is also implicated.^7^ Patients with a history of prior gastric surgery, such as partial gastrectomy including or excluding vagotomy, are at an increased risk of gastric bezoars, but patients with normal gastrointestinal anatomy and physiology can develop them as well.^3^ Common symptoms of gastric bezoars include nausea, vomiting, diarrhea, early satiety, epigastric discomfort, and weight loss. Complications of gastric bezoars include gastric outlet obstruction, ulceration, and perforation.^3^

The pathogenesis of intestinal bezoars typically involves collection of the offending undigested or inedible material in the intestines, usually in the ileum as it is difficult to pass the ileocecal valve as the material accumulates and hardens. Impaired peristalsis can play a role.^1^ Symptoms include abdominal pain that is usually generalized, as well as diarrhea. Complications of intestinal bezoars are intestinal obstruction, ulceration, bleeding, perforation, and hemorrhage.^3^

There is no specific method that currently exists to diagnose bezoars.^8^ This is most likely because they are rare, however, imaging with CT scan has been shown to play a major role in identification of bezoars after patients present with nonspecific gastrointestinal symptoms.^3^ Management typically involves attempts to dissolve bezoars with solutions, such as cellulase enzyme mixture, endoscopic removal if the prior fails, and surgery.^3^ Dissolving solutions or endoscopic removal are the primary management for gastric bezoars. However, surgery is usually necessary for intestinal bezoars, and for gastric bezoars that are too difficult to dissolve or remove endoscopically. A case of gastric bezoar that was initially being managed surgically by laparoscopy resulted in the surgery being converted to laparotomy as the bezoar was too difficult to remove.^9^

Surgery poses a risk for complications including minimally invasive surgeries. Such complications can involve the development of pelvic abscesses, which are infected exudate formed from liquefactive necrosis.^10^ Pelvic abscess is life‐threatening and can form after operative procedures, such as laparoscopies and laparotomies, for management of any condition requiring abdominal surgery.^11^ Surgery is usually reserved for when other management options have failed or in case of emergency. Hence, it is understood that when surgery can be prevented it is best to do so.

Geographic location may play a role in varying bezoar frequency especially as it relates to food cultures and phytobezoar formation. Diospyrobezoar is most found in communities that have persimmons in their regular diet. Southeastern United States, Japan, South Korea, Spain, Israel, and Turkey are locations that have high incidences of bezoars, and the inhabitants of these areas have a diet that includes frequent consumption of persimmons.^6^ For communities that consume persimmons on a regular basis, patient education should be updated to include information on bezoars, especially the diospyrobezoar subtype. Patients should be educated to consume persimmons only when ripened fully and to separate the pulp from the skin, to avoid the high tannin content. Individuals can be advised to reduce their intake of persimmons depending on their usual frequency of intake.

Trichobezoars are associated with underlying psychiatric disorders, such as developmental disorders, obsessive‐compulsive disorder, trichotillomania, and trichophagia, where individuals, most commonly young women, compulsively pulls strands of hair out and eats the hair; this is especially in times of increased stress.^12^ This eventually forms a bezoar composed of the ingested hair.^12^ Underlying behavioral disorders should be evaluated in patients who have the tendency to chew and swallow hair. For cases of bezoars where psychiatric conditions are identified, appropriate follow‐up with a psychiatric team is deemed essential to prevent recurrence. Prognosis is good if there is sufficient and successful psychiatric therapy to address the underlying behavioral disorder.^12^

Pharmacobezoars are associated with frequent and/or prolonged intake of medications and can cause symptoms of prolonged drug effects based on the medication make‐up of the bezoar. Medications that have been reported to cause pharmacobezoars include antacids, iron, aspirin, bulk laxatives, and extended‐release products such as theophylline and nifedipine.^13^ Sustained‐release products with unabsorbable components, such as insoluble cellulose acetate shell, are prone to pharmacobezoar formation.^13^ “Massive tablet ingestions, regardless of formulation, have caused pharmacobezoars”.^13^ Patients who take tablet supplements and medications should crush pills that are able to be crushed to prevent pharmacobezoars. They should be advised to first check with their health‐care provider to make sure that their medication is allowed to be crushed. Drinking lots of water when ingesting medication and supplements is recommended.

Lactobezoars occur mostly during early age. They are most common in infants who are fed infant milk formula with a high caloric density.^14^, ^15^ Adults have been reported to develop lactobezoars as well, but at a lower occurrence rate. These cases in adults are usually related to prior gastric surgery or overindulgence and frequent consumption of milk products, such as cheese or milk protein shakes.^16^ Lactobezoars can be prevented in infants by reducing the use of infant milk with enhanced caloric density. It can be prevented in adults by not consuming, at one time, high amounts of milk protein, such as in cheese. Moderation is the key for everyone, but especially for patients who have undergone gastric surgery.

Ingestion of tissue paper by patients with or without pica diagnosis has been associated with bezoars. A patient has been reported to swallow toilet paper as a means of dieting and developed multiple bezoars.^17^ A different patient with pica admitted to eating toilet paper due to cravings; this individual developed abdominal pain that was eventually found to be due to bezoars.^18^ Yet another pica‐associated case of intestinal obstruction occurred due to ingestion of Styrofoam balls.^5^

Pica should be addressed and treated to prevent occurrence or recurrence of bezoar due to tissue paper, foam, etc. In cases, such as this one where the patient was ingesting tissue paper, whether it be with or without pica diagnosis, the patient should be evaluated for any undiagnosed behavioral conditions, and similarly, underlying nutritional deficiencies should be addressed. Patients should be educated on the consequences that they are at risk of incurring if they continue to consume inedible material.

It is important to remind patients about the need to chew food properly. They should be educated to chew food about 10–30 times, or even more, depending on the hardness of the food until it is soft and has lost its consistency before swallowing. This is because a general risk factor that is usually not considered among citizens is not chewing food properly.^3^ This relates to the risk factors that are more common in the elderly, which include poorly fitting dentures or no teeth, which prevents them from chewing their food well. It is also advised to drink plenty of water to prevent bezoar formation or to avoid bezoar recurrence, as recurrence occurs in up to 20% of patients.^19^

In general, patient education at a regular doctor's visit does not usually include information on bezoars. Most people in the general population would not be able to give a simple definition of a bezoar. This is the objective of this case report; to encourage more conversations on this matter.

Considerations for incorporation into clinical practice can be to provide bezoar information to patients at their 6‐month or annual wellness visits, with the more frequent timing being geared towards populations that eat persimmons often. This information can be incorporated into diet and nutrition counseling. Printouts can be given as well for patients to read on bezoars. It is recommended to make the information on bezoars a routine reminder as part of general wellness so patients can have a better chance of preventing bezoars.

Such information can include the definition and types of bezoars, risk factors, and why it is necessary to evade these risk factors, for example, avoidance of unnecessary surgical procedures. Short bits of information on prior cases of bezoars and patient‐friendly images that are easily understood by the general population can be included for added perspective. Dissemination of information can occur through patient advocates, when necessary, which can be on the individual level or organizational level, where they will educate patients and encourage the entire team to make it a routine part of patient education.

It is not definite that the amount of bezoar cases will reduce by educating the general population; however, it is among the expected goals, as health education is a part of primary prevention. Education alone will not be sufficient if there are underlying developmental or psychiatric disorders, such as trichotillomania, and in such cases, it is imperative to address these underlying disorders with psychiatric consultation.^1^

## 4 CONCLUSIONS

This case report demonstrates that bezoars can cause gastrointestinal obstruction and can lead to unpredictable complications from management. It highlights the fact that bezoars are not to be ignored. Although rare, they can cause significant distress. Postsurgical complications, such as pelvic abscesses, can occur due to abdominal surgery itself. Therefore, a clear distinction must be made that although the patient developed pelvic abscess, this is a risk of surgery that can occur with other conditions as well, not just bezoars. However, surgery for obstructive bezoars can be avoided if bezoars can be prevented in the first place.

Bezoars should become a frequent topic of discussion among the general population. Many consequences may possibly be prevented if only information was disseminated more frequently, including the prevention of risks associated with surgery. When the unnecessary can be avoided by simply an increase in knowledge among the population and by enhanced patient education, it is then warranted to do so to avoid unnecessary health care visits. Further studies are suggested to investigate how to go about implementing bezoar information into patient education on a more widespread basis.

## AUTHOR CONTRIBUTIONS

**Shannon D. Powell:** Conceptualization; investigation; project administration; writing – original draft; writing – review and editing. **Nicholas Pereira:** Conceptualization; writing – review and editing.

## FUNDING INFORMATION

None declared. There are no financial agreements, funding, or grants for this research.

## CONFLICT OF INTEREST STATEMENT

None declared. We hereby declare that there is no conflict of interest regarding the publication of this paper, and we declare that the information given is true to the best of our knowledge.

## CONSENT

Telephone informed consent has been obtained from the patient's substitute decision‐maker (patient's father) with nurse as witness, followed by written informed consent signed by patient's father, for this case report and its publication.

## Data Availability

Data sharing is not applicable to this article as no datasets were generated or analyzed.

## References

[1] Tiago S , Nuno M , João A , Carla V , Gonçalo M , Joana N . Trichophagia and trichobezoar: case report. Clin Pract Epidemiol Ment Health. 2012;8:43‐45. 22675398 10.2174/1745017901208010043 PMC3367296

[2] Williams RS . The fascinating history of bezoars. Med J Aust. 1986;145:613‐614. 3540541

[3] Eng K , Kay M . Gastrointestinal bezoars: history and current treatment paradigms. Gastroenterol Hepatol (N Y). 2012;8:776‐778. 24672418 PMC3966178

[4] Claro M , Costa Santos D , Abreu Silva A , et al. When eating makes you sick – gastric stump obstruction caused by a phytobezoar. A case report and literature review. Int J Surg Case Rep. 2021;79:263‐266. 33485179 10.1016/j.ijscr.2021.01.034 PMC7820793

[5] Haji A , Sivakumar N , Bala P , Palit A . Eating styrofoam balls presented as internal obstruction‐PICA associated with bezoar: a rare case report and review of literature. Int Surg J. 2022;9:2034‐2036. https://www.ijsurgery.com/index.php/isj/article/view/9240

[6] Iwamuro M , Okada H , Matsueda K , et al. Review of the diagnosis and management of gastrointestinal bezoars. World J Gastrointest Endosc. 2015;7:336‐345. 25901212 10.4253/wjge.v7.i4.336 PMC4400622

[7] Dikicier E , Altintoprak F , Ozkan OV , Yagmurkaya O , Uzunoglu MY . Intestinal obstruction due to phytobezoars: an update. World J Clin Cases. 2015;3:721‐726. 26301232 10.12998/wjcc.v3.i8.721 PMC4539411

[8] Wang S , Yang X , Zheng Y , Wu Y . Clinical characteristics and indications for surgery for bezoar‐induced small bowel obstruction. J Int Med Res. 2021;49:1‐9. 10.1177/0300060520979377 PMC8162205 33445996

[9] Hennessy MM , Ivanovski I , Ó , Súilleabháin CB . Gastric ulceration and perforation secondary to large trichobezoar – a case report describing the role of magnetic resonance imaging in diagnosis. Int J Surg Case Rep. 2018;43:25‐28. 29438853 10.1016/j.ijscr.2018.01.004 PMC5814371

[10] Khaliq K , Nama N , Lopez RA . Pelvic Abscess. NIH NLM NCBI. Published January 2023. https://www.ncbi.nlm.nih.gov/books/NBK545292/

[11] Hadithi M , Bruno MJ . Endoscopic ultrasound‐guided drainage of pelvic abscess: a case series of 8 patients. World J Gastrointest Endosc. 2014;6:373‐378. 25132921 10.4253/wjge.v6.i8.373 PMC4133417

[12] Siddiqi J , Daous A , Shawosh Y . Trichobezoar due to psychiatric co‐morbidity: a rare case report. J Behav Health. 2017;6:70.

[13] Lung D , Cuevas C , Zaid U , Ancock B . Venlafaxine pharmacobezoar causing intestinal ischemia requiring emergent hemicolectomy. J Med Toxicol. 2011;7:232‐235. 21373970 10.1007/s13181-011-0144-8 PMC3151403

[14] Heinz‐Erian P , Gassner I , Klein‐Franke A , et al. Gastric lactobezoar – a rare disorder? Orphanet J Rare Dis. 2012;7:3. 22216886 10.1186/1750-1172-7-3 PMC3307440

[15] Castro L , Berenguer A , Pilar C , Gonçalves R , Nunes JL . Recurrent gastric lactobezoar in an infant. Oxf Med Case Reports. 2014;4:80‐82. 10.1093/omcr/omu031 PMC4399510 25988036

[16] Mayo J , Conrad R , Colgan B , Harjes W , Lim R . Ricotta cheese disease: Lactobezoar after roux‐en‐y gastric bypass. Society of American Gastrointestinal and Endoscopic Surgeons. Published 2017. https://www.sages.org/meetings/annual‐meeting/abstracts‐archive/ricotta‐cheese‐disease‐lactobezoar‐after‐roux‐en‐y‐gastric‐bypass/#:~:text=Conclusion%3A%20Lactobezoar%20is%20a%20rare,in%20this%20case%20ricotta%20cheese

[17] Goldman R , Schachter P , Katz M , Bilik R , Avigad I . A bizarre bezoar: case report and review of the literature. Pediatr Surg Int. 1998;14:218‐219. 9880754 10.1007/s003830050492

[18] Chronister E , Biggs D , Feldman D , Daramna Y . Bezoars: an interesting case of abdominal pain. JETem. Published January 16, 2020. https://jetem.org/bezoars/

[19] Setya A , Mendoza L , Fagan D . Fleece bezoar in a 6‐year‐old girl. Consultant360 Multidisciplinary Medical Information Network. Published September 2016. https://www.consultant360.com/articles/fleece‐bezoar‐6‐year‐old‐girl

